# Nanoscopic Supercapacitance Elucidations of the Graphene-Ionic Interface with Suspended/Supported Graphene in Different Ionic Solutions

**DOI:** 10.1021/acsami.4c16362

**Published:** 2025-01-13

**Authors:** Yu-Xuan Lu, Ming-Hsiu Tsai, Cheng-Yu Lin, Wei-Yen Woon, Chih-Ting Lin

**Affiliations:** 1 Graduate Institute of Electronics Engineering, National Taiwan University, Taipei 10617, Taiwan; 2 Department of Physics, National Central University, Jungli 32054, Taiwan; 3 Graduate School of Advanced Technology, National Taiwan University, Taipei 10617, Taiwan

**Keywords:** graphene supercapacitor, suspended graphene, graphene-ionic interface, solid−liquid interface, interfacial water arrangement

## Abstract

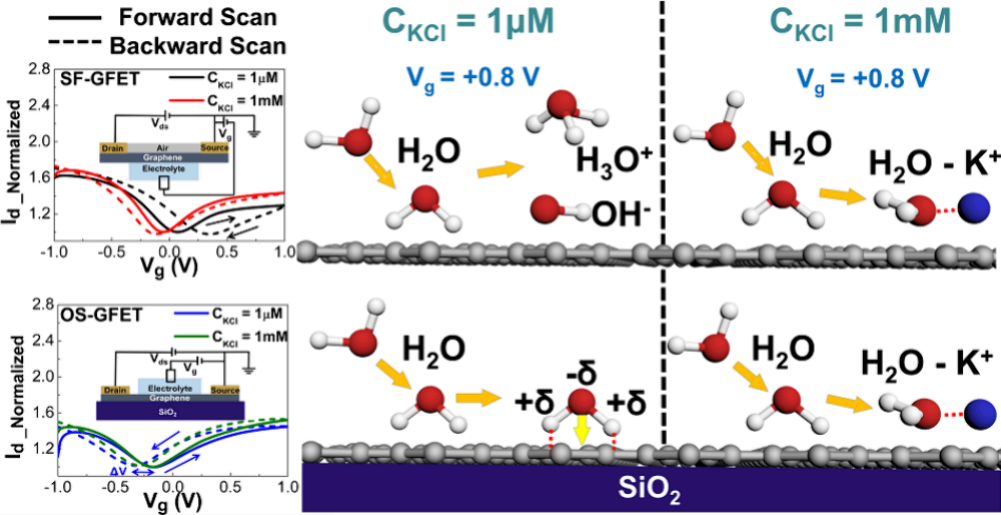

Graphene-based supercapacitors have gained significant attention due to their exceptional energy storage capabilities. Despite numerous research efforts trying to improve the performance, the challenge of experimentally elucidating the nanoscale-interface molecular characteristics still needs to be tackled for device optimizations in commercial applications. To address this, we have conducted a series of experiments using substrate-free graphene field-effect transistors (SF-GFETs) and oxide-supported graphene field-effect transistors (OS-GFETs) to elucidate the graphene-electrolyte interfacial arrangement and corresponding capacitance under different surface potential states and ionic concentration environments. For SF-GFET, we observed that the hysteresis of the Dirac point changes from 0.32 to −0.06 V as the ionic concentration increases. Moreover, it results in the interfacial capacitance changing from 4 to 2 F/g. For OS-GFET, the hysteresis of the Dirac point remains negative (−0.15 to −0.07 V). Furthermore, the corresponding capacitance of OS-GFET decreases (53–16 F/g) as the ionic concentration increases. These suggest that the orderly oriented water structure at the graphene–water interface is gradually replaced by ionic hydration clusters and results in the difference of capacitance. The relationship between Dirac-point hysteresis value and ionic concentration can be modeled by using the first-order Hill equation to obtain the half occupation value (*K* = 1.0131 × 10^–4^ for KCl solution and *K* = 6.6237 × 10^–5^ for MgCl_2_ solution). This also agrees with the variances of two minerals in ion hydration within the inner water layer at the interface. This work illustrates the influence of interfacial nanoscale arrangement on interface capacitance formation and layout implications for the development of supercapacitors.

## Introduction

In response to the needs of modern society and growing ecological concerns, it is crucial to develop new, low-cost, and environmentally friendly energy storage systems.^1^ Supercapacitors, known for their exceptional power density and extended lifespan, are attracting significant research interests for diverse applications, including energy storage systems, power backup solutions, renewable energy integration, and automotive systems.^2−5^ At the same time, nanostructured materials are widely employed in supercapacitors, encompassing carbon-based substances, metal oxides, and conductive polymers.^6−8^ Among these nano materials, graphene is a promising electrode material for energy storage.^9,10^ It has exceptional electrical conductivity and strength, coupled with its remarkably high specific surface area of up to 2675 m^2^/g, providing favorable conditions for its utilization in high-performance supercapacitors.^10−12^ The outstanding capacitance performance of graphene-based supercapacitors is increasingly being recognized.

According to previous research, the intrinsic capacitance of monolayer graphene with ionic solution is around 1 μF/cm^2^, and the capacitance value increases with the number of layers.^13^ A supercapacitor based on graphene electrodes chemically modified with nitrogen or graphene produced through the exfoliation and reduction of graphene oxide exhibits higher capacitances.^14,15^ Despite numerous efforts to optimize these properties, achieving the desired supercapacitor performance with graphene-based materials remains challenging due to inherent material characteristic limitations. Addressing these obstacles calls for innovative strategies to enhance material traits and surpass constraints such as the compatibility between intrinsic graphene sheets and ionic liquids. On the other hand, in previous experimental studies of 2D material-electrolyte characteristics, most graphene-related devices were situated on a substrate.^16−19^ As a result, these studies encountered alterations due to substrate effects at the graphene-solution interface, leading to changes in surface potentials.^20,21^

To address the fundamental difference between previously experimental and theoretical results, in this work, we prepare two kinds of graphene field-effect transistors, i.e., suspended (SF-GFET) and oxide-supported (OS-GFET), with one side of graphene in contact with electrolytes. The interfacial ion arrangement at the inner layer is verified successfully by experiments for the first time and shows consistency with the simulation results. Simultaneously, it is noteworthy that the surface potential and ion concentration exert notable effects on the formation of interfacial capacitance.

## Experimental Section

### Experimental Device Structure and Characteristics

**Figure 1:**
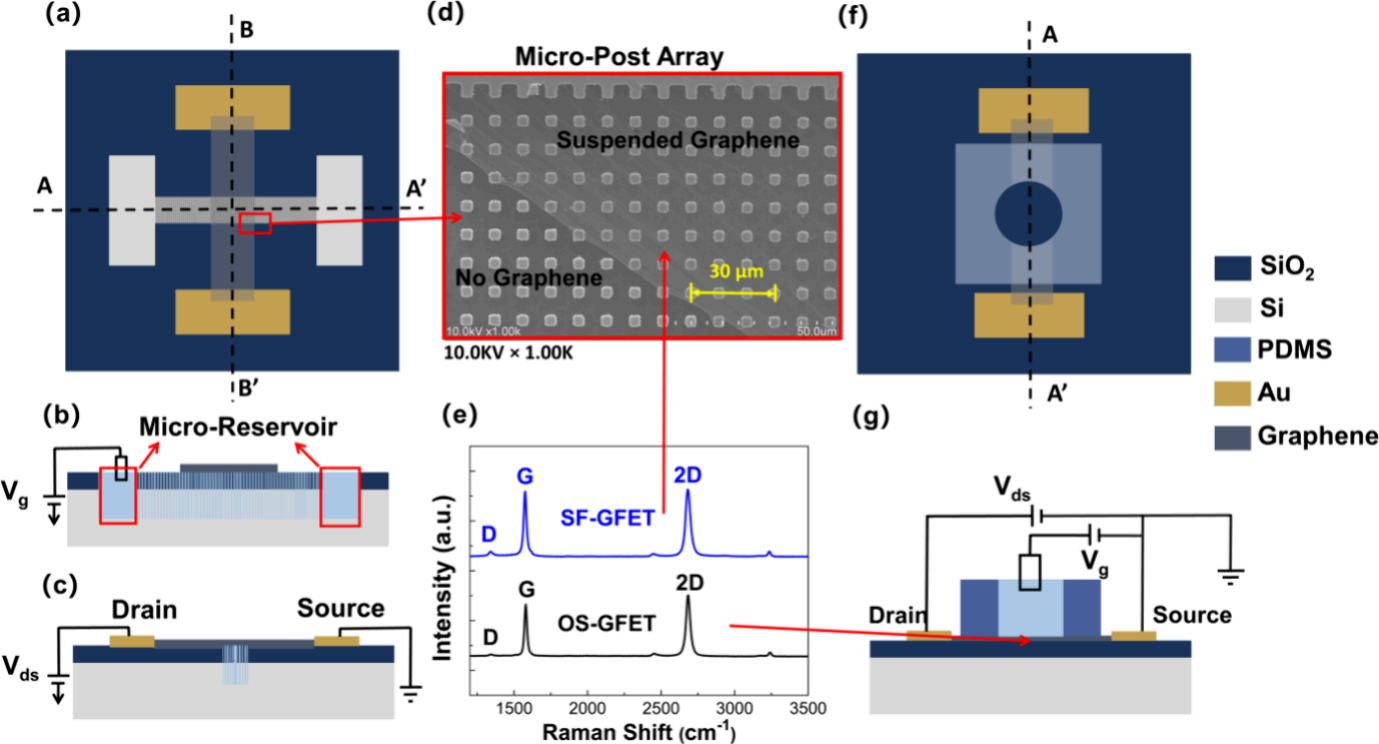
Schematic view of the SF-GFET/OS-GFET device and measurement diagram. (a) Top view of the SF-GFET device. (b) View at the A–A′ direction of the SF-GFET device cross section. (c) View at the B–B′ direction of the SF-GET device cross section. (d) SEM image of graphene suspended on the micropost array in the SF-GFET device. (e) Raman spectra of suspended graphene in the SF-GFET device (blue line) and supported graphene of the OS-GFET device (black line). (f) Top view of the OS-GFET device. (g) View at the C–C′ direction of the OS-GFET device cross section.

Figure 1a shows a schematic of the SF-GFET device. A microfluidic channel with the micropost array is fabricated to make graphene suspended and contact with electrolyte in the microfluidic channel. The micropost array comprises 5 μm square pillars with a 5 μm period. Microreservoir regions are present at both ends (A–A′) of the channel for the addition of electrolytes, as shown in Figure 1b. Graphene spans across the microfluidic channel and builds an electrical channel between the source and drain (B–B′), as shown in Figure 1c. The top SEM view of graphene suspended on the micropost array in the SF-GFET device is shown in Figure 1d. This figure clearly shows the structure of the micropost array and the presence of large-area graphene with excellent quality. The SEM image of SF-GFET with upright and tilted angles is shown in Figure S1a, which demonstrates the graphene's successfully suspended state. Figure 1e displays the Raman spectra of SF-GFET in the blue line and supported graphene of the OS-GFET device in the black line; the prominent characteristics of the G and 2D peaks prove the existence of graphene. The *I*_D_/*I*_G_ ratio of SF-GFET (0.074) and OS-GFET (0.029) indicates the high quality of material.^22^ Furthermore, the *I*_2D_/*I*_G_ ratios of SF-GFET (1.18) and OS-GFET (1.03), along with the FWHM of *I*_G_ (23.08 cm^–1^ for SF-GFET and 21.85 cm^–1^ for OS-GFET), suggest bilayer graphene with high crystallinity in both devices.^23−25^ A more comprehensive regional Raman analysis confirms the superior quality of graphene (Supporting Information: The Characteristics of the SF-GFET Device and the OS-GFET Device). The corresponding optical images of SF-GFET and OS-GFET also demonstrate the large area of high-quality graphene, as illustrated in Figure S1b. In contrast to SF-GFET, Figure 1f shows that the OS-GFET device contains a PDMS (polydimethylsiloxane) reservoir placed on top of the graphene channel to apply the gate voltage and prevent solution leakage. The gate voltage is applied to ionic solution through the Ag/AgCl electrode (C–C′), as shown in Figure 1g. The detail fabrication process can be shown in Figure S2.

### Fabrication of the Fluidic Channel in the SF-GFET Device Substrate

The detailed fabrication process of SF-GFET is illustrated in Figure S2, which mainly consists of bilayer graphene and suspended substrate fabrication. Bilayer graphene fabrication is a critical process. The monolayer graphene film is grown on the copper foil by chemical vapor deposition (CVD) and then separated by electrical bubble delamination with the support of a PMMA-protective film. To establish the bilayer graphene, the monolayer graphene is physically stacked together in a deionized (DI) water environment and the water is removed by spinning and baking. Further overlaying and separation also rely on the support of the PMMA sheet. To fabricate the microchannel structure, on the other hand, the photoresist is spun onto a SiO_2_ (300 nm)/Si substrate and a microfluidic pattern is generated by photolithography. After dry etching the SiO_2_ with a thickness of 300 nm and Si layers with a depth of 30 μm, the microfluidic trench can be used to store aqueous solution. Then, Au electrodes adjacent to the microfluidic channel are deposited through evaporation. Afterward, the bilayer graphene film is transferred across the trench. Finally, SF-GFET is immersed in acetone for 24 h to remove the PMMA layer. Then, the device is submersed in isopropanol (IPA) and DI water for 5 min to cleanse any residues.

### Electrical Measurement of the GFET Device

The electronic transport characteristics under an aqueous environment were measured by an Agilent Semiconductor B1500A analyzer. There are three electrical channels, i.e., drain (d), source (s), and gate (g), in our experimental setup. The drain-source voltage (*V*_ds_) is fixed at 0.1 V to get the channel current where the source is grounded. Then, the gate voltage (*V*_g_) scans from −1 to +1 V (forward scanning process) and then reverses from +1 to −1 V (backward scanning process) with a specific step rate. In our measurement setup, it should be noted that *V*_g_ changes and then retains the changed value for stabilization. This process is repeated for the next voltage step. This time is much longer than the molecule dissociation and reorientation time (∼ps). Thus, current values are measured at a steady state. Then, the change in charge at each steady-state condition can be calculated through Δ*Q* = Δ*I* × Δ*t*, where Δ*I* is the change in current (Δ*I* = *I*_d_ + *I*_s_ + *I*_g_). According to Δ*Q* = *C* × Δ*V*, where *V* is the applied gate voltage and *C* is the capacitance, the capacitance value can be calculated. Through this method, we can identify the maximum capacitance during the forward scanning process. Furthermore, the measurements were conducted in electrolyte solutions with concentrations ranging from 1 μM to 1 mM. To ensure consistent analysis and avoid concentration-induced effects, the experiments progressed systematically from lower to higher ionic concentrations.

## Results and Discussion

### Comparison of the Hysteresis Effect

**Figure 2:**
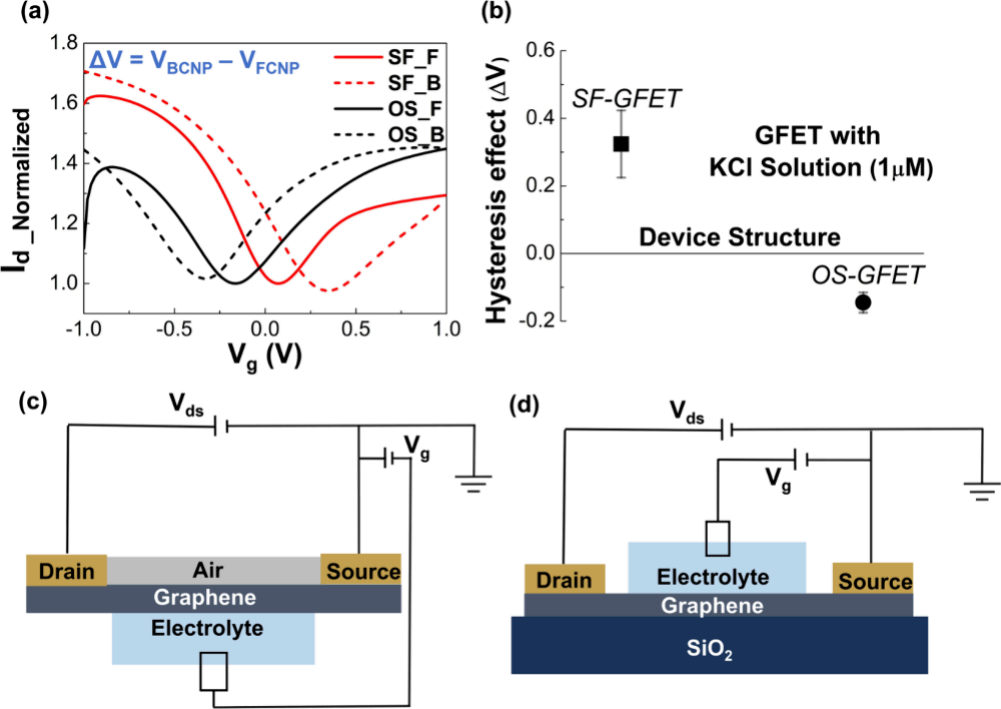
Comparison of graphene transport behavior of SF-GFET and OS-GFET in 1 μM KCl electrolyte. (a) *I*_d_–*V*_g_ curve (SF_F: SF-GFET in the forward scan; SF_B: SF-GFET in the backward scan; OS_F: OS-GFET in the forward scan; and OS_B: OS-GFET in the backward scan). (b) Experimental results of the hysteresis effect (Δ*V*) of SF-GFET and OS-GFET. (c) Schematic of the experimental setup of the SF-GFET. (d) Schematic of the experimental setup of OS-GFET.

The Dirac-point hysteresis responses of both SF-GFET and OS-GFET devices are measured individually by contacting with electrolytic solutions of KCl concentration at 1 μM, as shown in Figure 2a. The drain current is recorded as *V*_ds_ = 0.1 V with a scanning gate voltage (*V*_g_). Moreover, the drain current, *I*_d_, is normalized to mitigate the variability across different devices. The solid line (SF_F and OS_F) corresponds to the forward-scanning direction of the gate voltage (from −1 to +1 V), and the dotted line (SF_B and OS_B) represents the backward-scanning direction of the gate voltage (from +1 to −1 V). The Dirac-point hysteresis effect can be defined as Δ*V* = *V*_BCNP_ – *V*_FCNP_, where *V*_FCNP_ and *V*_BCNP_ denote the charge neutrality points of the forward-scanning and backward-scanning processes, respectively. The SF-GFET (SF_F and SF_B), i.e., the red line in Figure 2a, exhibits a positive hysteresis effect, with Δ*V* around 0.28 V. In contrast, the OS-GFET (OS_F and OS_B), i.e., the black line in Figure 2a, demonstrates a negative hysteresis effect, with Δ*V* around −0.16 V. The discrepancy in hysteresis signs emphasizes the substantial impact of the oxide substrate on the configuration of the graphene–electrolyte interface. The further statistical results showed a hysteresis effect of 0.32 ± 0.099 V for SF-GFET (*n* = 5) and −0.15 ± 0.030 V for OS-GFET (*n* = 4), as depicted in Figure 2b. This indicates that carrier trapping is more obvious for the intrinsic graphene–water interface, i.e., SF-GFET. Meanwhile, the hysteric result exhibits distinct interfacial evolution when undergoing the *V*_g_ scanning. The schematics of the electrical connection model of SF-GFET and OS-GFET are represented in Figure 2c and 2d, respectively. Owing to air adsorbates hardly influencing the hysteresis effect, the influence of air can thus be neglected in our experiment structure.^26^ The difference mainly comes from the influence of the surface conditions. In previous simulation works, it should be noted that the ionic concentration is exceptionally high, e.g., hundreds of millimolars or a few molars.^27−32^ Our experiments mainly focus on measuring the hysteresis effect of electrolyte solution within the range of 1 μM–1 mM. Therefore, field-effect transport mainly comes from inner-layer interfacial evolution.

### Effect of Salt Concentration at the Interface

**Figure 3:**
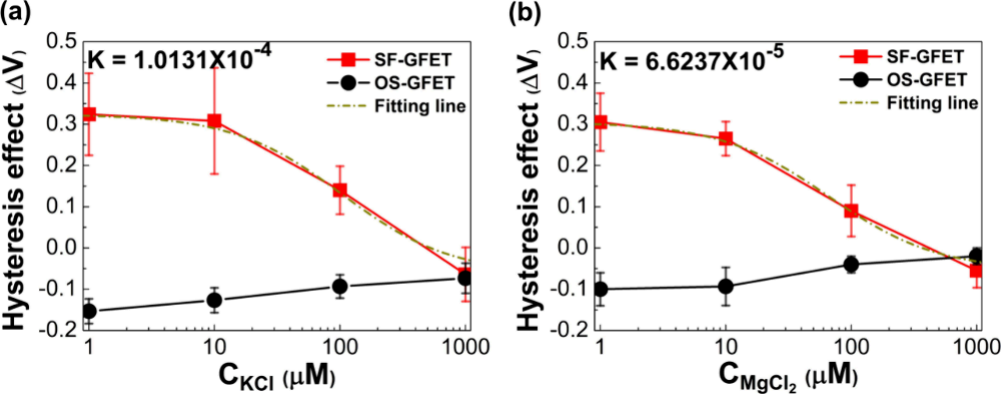
Comparison of graphene transport behavior of SF-GFET and OS-GFET in electrolytes with concentrations at 1, 10, and 100 μM. (a) Hysteresis effect (Δ*V*) of SF-GFET and OS-GFET in KCl aqueous solution. (b) Hysteresis effect (Δ*V*) of SF-GFET and OS-GFET in MgCl_2_ aqueous solution.

To further investigate the hysteresis effect of devices with respect to different valence ions (K^+^ and Mg^2+^) with different concentrations, we choose KCl and MgCl_2_ as electrolytic solutions of controlled concentrations (1 μM, 10 μM, 100 μM, and 1 mM). In this investigation, the electrolytic concentration is tested from a low to a high concentration to ensure consistent analysis and avoid potential concentration-induced effects during testing. For the case of SF-GFET with KCl aqueous solution at different concentrations, the hysteresis values are positive (0.32, 0.31, and 0.14 V) at 1, 10, and 100 μM, respectively. Moreover, it is negative (−0.06 V) at 1 mM. In comparison, for the case of OS-GFET with KCl aqueous solution at various concentrations, the hysteresis values are negative (−0.15, −0.13, −0.09, and −0.07 V) at 1 μM, 10 μM, 100 μM, and 1 mM, respectively, as shown in Figure 3a. The hysteresis value shows a negative relationship with the ionic concentration for SF-GFET, and the hysteresis value shows a positive relationship with the ionic concentration for OS-GFET. The changed sign at 1 mM SF-GFET shows that the origin interface is disrupted by ions at high ionic concentrations. Correspondingly, carrier transport results of SF-GFET and OS-GFET devices in MgCl_2_ aqueous electrolyte show a similar trend, as illustrated in Figure 3b. According to the results, the relationship between the hysteresis value of SF-GFET and the ionic concentration can be written as the following equation.
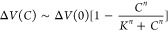
1where Δ*V*(*C*) in eq 1 corresponds to the fitted hysteresis effect under a certain concentration *C*, Δ*V*(0) is the measured hysteresis value with DI water, and *n* and *K* are constants. The fitting result is characterized by the first-order Hill’s equation, indicating that the ions’ adsorption with dissociable water at the inner layer exhibits characteristics of nonadditive and nonresistive.

According to the fitting result, *K*_KCl_ = 1.0131 × 10^–4^ for KCl electrolytic solution and *K*_MgCl2_ = 6.6237 × 10^–5^ MgCl_2_ electrolytic solution. This shows that the ionic concentration produces half of the occupation. Moreover, the significant decrease only happens in the concentrations from 1 to 1000 μM. Further addition (concentration ≥1000 μM) or subtraction (concentration ≤0.1 μM) of cations does not cause a significant change in hysteresis value. As the ionic environment concentration in previous simulation studies is exceptionally high, e.g., over hundreds of millimolars, the cation may saturate at the subsurface layer,^31,32^ and the Helmholtz layer dominates the interfacial characteristics.^33,34^ These do not facilitate an understanding of local surface changes.

**Figure 4:**
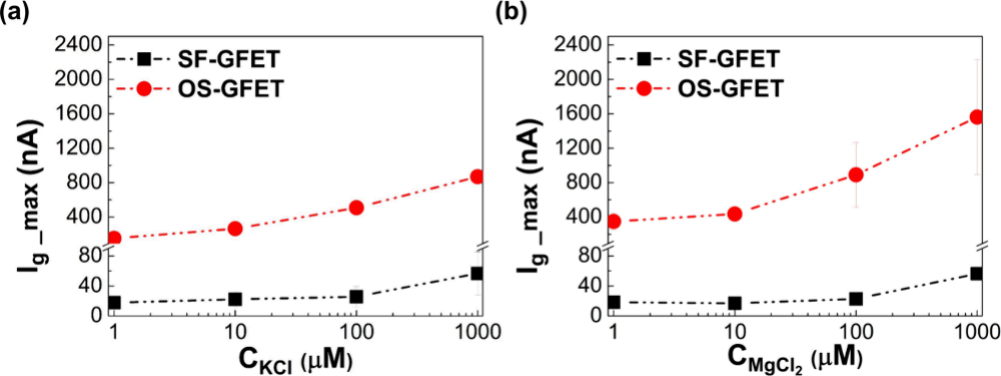
Comparison of graphene transport behavior of SF-GFET and OS-GFET in electrolytes with concentrations at 1, 10, and 100 μM. (a) Maximum gate currents of SF-GFET and OS-GFET in a KCl aqueous solution. (b) Maximum gate currents of SF-GFET and OS-GFET in MgCl_2_ aqueous solution. The black rectangles and red circles show the value corresponding to the maximum gate currents of SF-GFET and OS-GFET, respectively.

It is intriguing to understand the detailed adsorbed configuration to determine the hysteresis process under the ionic environment. In Figure 4, it can be noted that the gate current (*I*_g_) of SF-GFET behaves similarly at relatively low concentrations (1, 10, and 100 μM) and increases obviously at a high concentration (1 mM). The maximum gate currents of KCl and MgCl_2_ electrolytes with different concentrations are shown in Figure 4a and 4b, respectively. The gate current of OS-GFET keeps increasing as the environmental concentration increases. As the ion concentration increases, the interface undergoes rearrangement, resulting in the enhancement of longitudinal transport. The maximum gate current shows a positive relationship with the concentration.

### Interfacial Configuration at a Steady State

When graphene interfacial configurations are discussed, it is important to consider the interfacial arrangement at a steady state. Both water and ions play crucial roles in interfacial configurations on the graphene surface. On the one hand, the interfacial water molecules would be reordered when a hydrophobic surface comes into contact with an aqueous solution.^33−36^ Partial interfacial water molecules are dissociated at the graphene–water interface and form two ions (H_3_O^+^ and OH^–^). Moreover, majority of water molecules around OH^–^ and H_3_O^+^ rearrange their OH group parallel to the surface.^33^ These water molecules and dissociated water molecules form a 2D hydrogen-bond network structure (2D HBNS).^37^ This hyper-coordinated structure prevents the recombination of two water ions (H_3_O^+^ and OH^–^) and causes the formation of a layered structure (i.e., first water layer (FWL) and second water layer (SWL)) close to the surface.^38^ Intense layering at the graphene surface is characterized by two dense subsurface layers and nearly constant above 8 Å from the surface.^28,38,39^ On the other hand, ion-graphene attachment at the interface is related to ionic characteristics (such as charge, size, and concentration) and hydration energy (related to charge, size, charge, and polarizability).^27−30,40−42^ Compared with large and less charged ions, small and highly charged ions induce a significantly higher degree of polarization in the delocalized electrons within the graphene sheet. This results in different binding energies.^42^ Specifically, the adsorption energy strengths between ion and π are around −29, −250, and −11 kcal/mol for K^+^–π, Mg^2+^–π, and Cl^–^–π, respectively.^30,41^ At the same time, the hydration energies of distinct ions also exhibit significant differences. For instance, the interaction between K^+^ and water is much weaker than that between Mg^2+^ and water.^40^ When the ions approach the graphene interface, as a consequence, K^+^ partially dehydrates, and Mg^2+^ preserves their first hydration shells.^27^ In addition, adsorbed Cl^–^ at the graphene interface also prefers to keep the first hydration shell intact. According to previous simulation results, the semihydrated potassium ions are located at around 3 Å from the graphene surface, the chloride ion layer lies at around 4 Å away from the graphene, and the magnesium ions are located at around 4.3 Å from the graphene surface.^27^ The positions of these ions are all within the FWL, which is positioned around 0–5 Å at the graphene interface.^34,35^ When graphene is supported by a SiO_2_ substrate, e.g., OS-GFET, instead of freestanding in air, the interface is strongly affected by the underlying substrate. The SiO_2_ substrate makes the graphene surface hydrophilic.^33,43,44^ Then, water partially points to the surface and forms hydrogen-bonding networks at the hydrophilic surface.^45^ Meanwhile, more ions and water accumulate at the OS-GFET surface.

**Figure 5:**
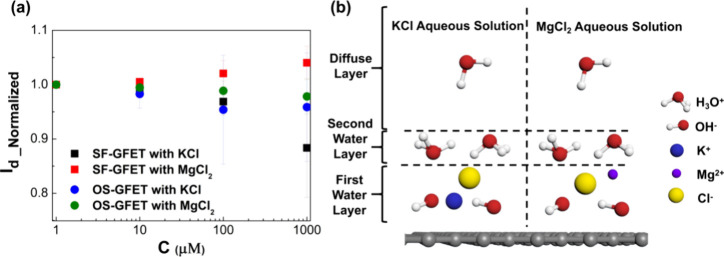
(a) Drain current of SF-GFET with constant voltage in KCl and MgCl_2_ electrolytes with concentration at 1 μM, 10 μM, 100 μM, and 1 mM. (b) Distribution of ions at the surface in potassium chloride aqueous solution and magnesium chloride aqueous solution. For clarity, it is represented exclusively in an ionic state.

In order to verify the variations in ion distribution at the graphene surface in a steady state, we monitored the *I*_d_ of the SF-GFET in different ionic aqueous environments by applying a constant channel voltage (*V*_ds_) of 0.1 V. The current remains stable at a steady level after the interfacial structure stabilizes. As shown in Figure 5a, *I*_d_ of SF-GFET with potassium chloride and magnesium chloride aqueous solutions exhibited opposite trends in response to an increasing concentration. This indicates that the charge distribution of ions at the solid interface has opposite polarities at a steady state. This is consistent with the results of previous simulations. As depicted in Figure 5b, potassium ions are located closer to the graphene surface due to their adsorption energy, positioned on the inner side of the chloride ions. More precisely, the significantly dissociated hydroxide ions (OH^–^) and potassium ions (K^+^) are captured inside the primary water layer. Chloride ions (Cl^–^) are repelled from the interface and reside at the fringe of the foremost water layer with an electrostatic attraction effect. Corresponding hydronium ions (H_3_O^+^) are found in the subsequent water layer. In contrast, magnesium ions exhibit the opposite behavior, positioning themselves on the outer side of the chloride ions. It is noteworthy that the *I*_d_ of the OS-GFET drops with decreasing concentrations of potassium chloride and magnesium chloride aqueous solutions, as shown in Figure 5a. The similar trends in current variation between the two solutions suggest that the surface potential alters the arrangement of surface ions and facilitates the proximity of magnesium ions to the graphene surface. Unlike the significant current change observed in SF-GFET, the current variation in OS-GFET is relatively small. This may come from the interference of the SiO_2_ substrate.^46,47^

### Interfacial Evolution under Dynamic Electrical Modulation

Dynamic electric modulation induces dynamic changes in the interfacial arrangement, thus facilitating our deeper exploration of the interface component. Since the hysteresis value of SF-GFET is positive at low concentrations (1, 10, and 100 μM) and negative at a high concentration (1 mM), we further explore the distinction between interfacial changes by comparing the carrier transport behavior with electrolyte concentration at a low concentration (1 μM) and a high concentration (1 mM). Hysteresis variation of SF-GFET and OS-GFET with KCl and MgCl_2_ aqueous solutions with concentration show a similar trend; we utilize potassium chloride aqueous solution as an example to delve into the details.

**Figure 6:**
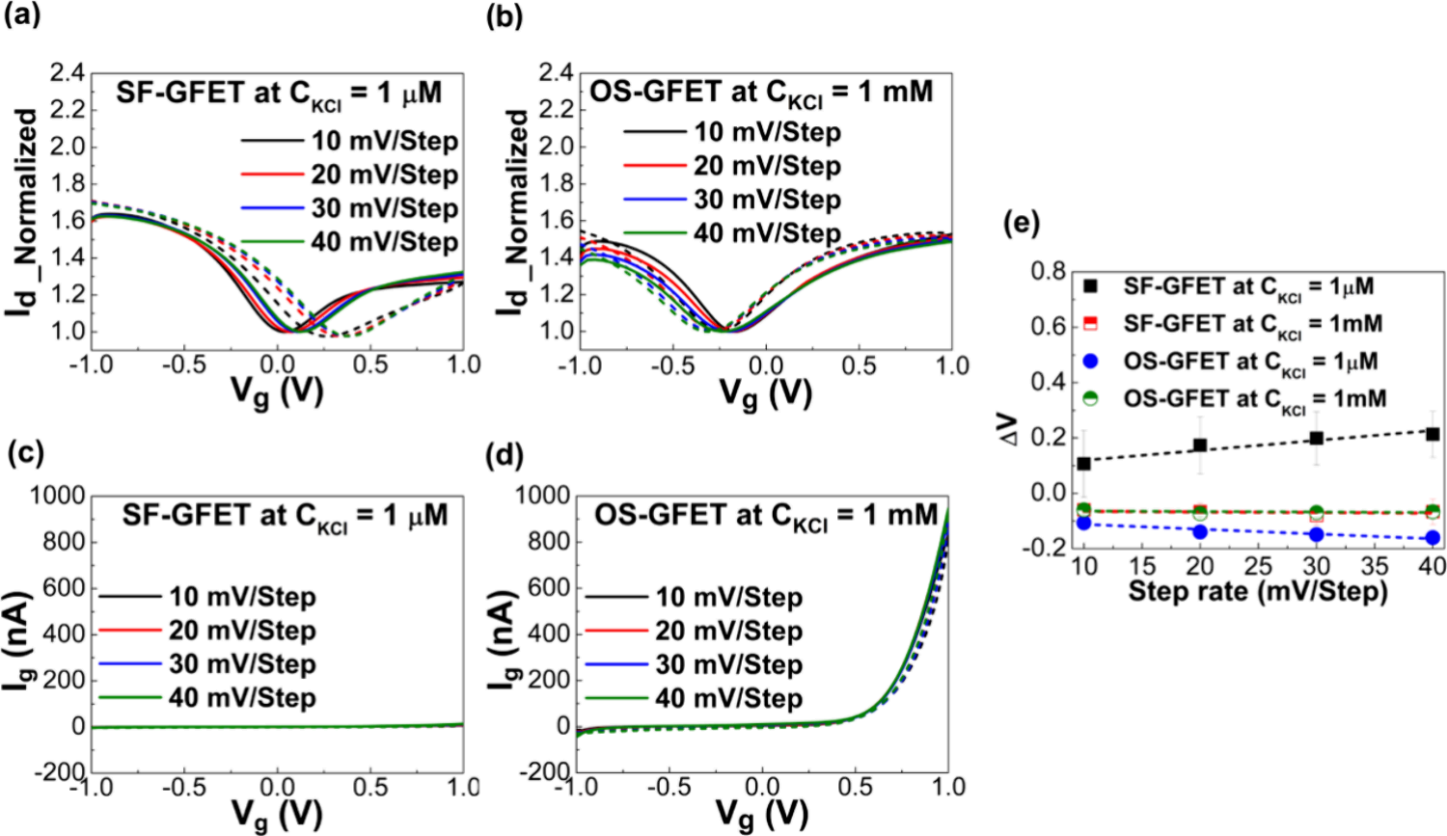
Drain current behavior (*I*_d_) and gate current behavior (*I*_g_) of GFET with changed gate voltages and different step rates: (a) *I*_d_ of SF-GFET contact with 1 μM KCl electrolyte solution; (b) *I*_d_ of OS-GFET contact with 1 mM KCl electrolyte solution; (c) *I*_g_ of SF-GFET contact with 1 μM KCl electrolyte solution; (d) *I*_g_ of OS-GFET contact with 1 mM KCl electrolyte solution; (e) hysteresis effect of SF-GFET and OS-GFET with increasing gate-voltage steps at 1 μM and 1 mM KCl aqueous concentration.

To explore the interfacial transformation of SF-GFET with one-side electrolyte solution as 1 μM KCl aqueous solution and OS-GFET with 1 mM KCl aqueous solution, the hysteresis behavior through the graphene field-effect current (*I*_d_) and the conducting path through the gate current (*I*_g_) with different gate voltage step rates are monitored by experiments, as shown in Figure 6a,b and Figure 6c,d, respectively. The electrical results of SF-GFET with one-side electrolyte solution as 1 mM KCl aqueous solution and OS-GFET with 1 μM KCl aqueous solution are shown in Figure S3. In these figures, *I*_d_ exhibits variations during the *V*_g_ scanning process, indicating dynamic changes in the interfacial configuration. Conversely, changes in *I*_g_ do not display a pronounced correlation with changes in *I*_d_. This suggests that the interfacial capacitive gating effect primarily drives channel doping. The relative shift in the hysteresis value at different stepping rates is illustrated in Figure 6e. The hysteresis effect increases with the rising step rate in the 1 μM environment, while the hysteresis value fluctuates within a small range in the 1 mM environment. These contrasting trends between low and high concentrations signify variations in the dominant factors driving interface alterations. This phenomenon is attributed to the competitive interplay between 2D HBNS and ionic hydration at hydrophobic surfaces, as well as the reorientation of water molecules toward hydrophilic surfaces or ionic clusters.^48^ As depicted in Figure 6c, the gate current (*I*_g_) of SF-GFET remains relatively consistent throughout the *V*_g_ scanning process. In contrast, unlike the behavior observed in SF-GFET, the gate current *I*_g_ of OS-GFET exhibits diode-like characteristics, as illustrated in Figure 6d. Notably, the evolving surface structure manifests distinct disparities under negative and positive gate voltage regions. A conductive pathway between the OS-GFET graphene and the solution-gate electrode is created at positive gate voltages, specifically gate voltages over 0.6 V. Meanwhile, the conducting gate current of OS-GFET increases in correlation with the concentration. Both the gating effect and the conductive pathway contribute to the interfacial state.^49,50^

In the dynamic evolution of electrical modulation, ions experience vertical diffusion, migrating closer or farther from the graphene interface in tandem with dehydration or hydration processes.^27^ This dynamic change presents the potential to induce the reorientation of interfacial water molecules and the subsequent formation of ionic hydration with these molecules,^45^ setting the stage for a competition between the hydrogen-bond structure within the water hydration cluster.^27,28,51,52^ At hydrophobic surfaces, i.e., SF-GFET, the formation of a hyper-coordinated structure results in a notable decrease in the self-diffusivity of hydroxide ions (OH^–^) compared to hydronium ions (H_3_O^+^), a phenomenon accentuated within the 2D HBNS structure. Additionally, water molecules exhibit faster diffusion rates than all ions at the interface, thereby influencing the dynamics of interfacial water.^28,53^ Furthermore, studies have shown that Cl^–^ and K^+^ display comparable in-plane diffusivity, thus having little impact on interface alterations within the plane.^28^ In this experiment, therefore, changes in ion composition at the hydrophobic interface are primarily driven by fluctuations in the gate potential and electrostatic attraction. Conversely, at hydrophilic surfaces, i.e., OS-GFET, water molecules exhibit relatively weak hydrogen bonding. This collective dipole behavior is readily modulated by external electric fields.^38,54^ Moreover, the higher electronegativity at the hydrophilic interface contributes to a greater concentration of surface cations compared with hydrophobic surfaces.

**Figure 7:**
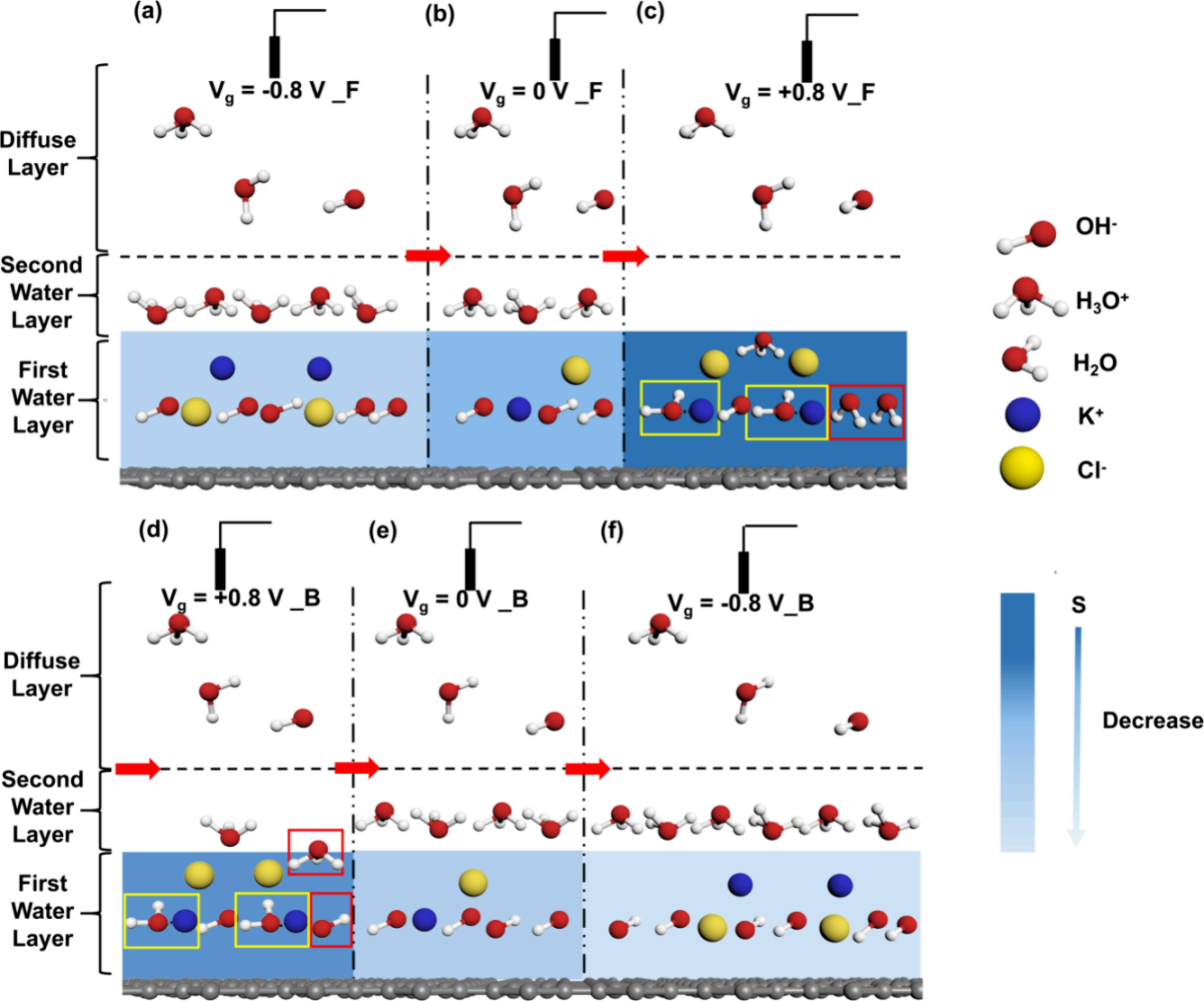
Schematic of interfacial evolution of SF-GFET with 1 μM KCl electrolyte solution with different applied gate voltages. The right color bar shows the population degree of dangling water molecules “S” in the first water layer. The dangling water (“S”) distribution intensifies for dark blue and diminishes for light blue. For clarity, 2D HBNS and ionic hydration clusters are represented exclusively in an ionic state: (a) *V*_g_ = −0.8 V at the forward scan path. (b) *V*_g_ = 0 V at the forward scan path. (c) *V*_g_ = 0.8 V at the forward scan path. (d) *V*_g_ = 0.8 V at the backward scan path. (e) *V*_g_ = 0 V at the backward scan path. (f) *V*_g_ = −0.8 V at the backward scan path.

Drawing from the preceding discussions, we clarify the connection between water molecules’ arrangement at interfaces and ions’ distribution configuration within those interfacial water layers. Figure 7 portrays the schematics of interfacial evolution at the SF-GFET interface with one-side 1 μM KCl aqueous solution under electrical modulation as the gate-voltage-scan process. 2D HBNS weakens as the electric field changes from directing-away-from-surface to directing-toward-surface.^33^ This causes part of water in 2D HBNS turn to form a hydration shell with the surrounding ions.^28^ At the same time, part of water in 2D HBNS becomes dangling water molecules,^33,55^ as indicated by the “S″ on the right side of the bar in Figure 7. These dangling water molecules’ OH groups tend to point toward the surface, and their reorientation time is much slower than that of typical molecules.^33,55,56^

To illustrate the detailed evolution process, we explain the state transitions by interpreting specific voltage conditions. Initially, *V*_g_ starts from the negative voltage, creating a significant electric field directed away from the surface. This negative electric field strengthens the 2D HBNS. At the same time, the negative electric field increases the concentration of ions at the interface.^33,57^ Meanwhile, the interfacial chloride ions (Cl^–^) move toward the surface,^42^ while potassium ions (K^+^) move away. Due to electrostatic attraction, K^+^ ions are situated close to Cl^–^ ions within the FWL.^28^ Because of the low concentration of ions, the dominance of the 2D HBNS leads to a more negatively charged interface and the introduction of holes in graphene, as shown in Figure 7a. As *V*_g_ moves toward 0 V at the forward scanning process, the negative electric field decreases. This causes the 2D HBNS to weaken, resulting in less negative charge at the interface. Simultaneously, Cl^–^ ions move away from the surface and spread within the inner side of the FWL, while K^+^ ions move closer to the surface driven by the ion−π interaction.^42^ Consequently, electrons become the primary carriers of graphene, instead of holes. The resulting distribution at the interface is depicted in Figure 7b. As *V*_g_ is further increased to positive voltage, the 2D HBNS weakens significantly, leading to a decrease in dissociation.^33^ Thus, as illustrated in Figure 7c, there is a notable increase in the number of water molecules. The accumulations of K^+^ and Cl^–^ at the FWL increases. Despite the low concentration of ions in solution, the abundance of interfacial water molecules facilitates ionic hydration, as indicated by the yellow box in Figure 7c. Additionally, the insufficient number of ions fails to fully deplete water molecules; this facilitates the formation of dangling water molecules under the applied electric field (as indicated by the red box in Figure 7c), contributing to the electronic doping of graphene. During the backward scanning process (*V*_g_ scanning from positive to negative), interfacial evolution proceeds more quickly than the forward scanning rate due to the highly ordered arrangement of dangling water molecules, as indicated by the red box in Figure 7d. Consequently, the water redirected by ions remains unaltered, as illustrated by the yellow box in Figure 7d. The distribution of K^+^ and Cl^–^ closely resembles that at *V*_g_ = +0.8 V, as depicted in Figure 7d. As *V*_g_ approaches 0 V during the backward scanning process, as shown in Figure 7e, the concentration of ions (H_3_O^+^ and OH^–^) increases at the interface; this results in strengthening of the 2D HBNS. This interfacial configuration change leads to higher negative charge at the interface. This is the reason for the hysteresis effect. Ultimately, as *V*_g_ becomes increasingly negative in the backward process, the dissociation rate of water molecules exceeds that of the forward process, leading to further strengthening of the 2D HBNS. This results in a predominance of backward-path hole current over forward-hole path current. Meanwhile, Cl^–^ aggregates tightly toward the surface, with K^+^ ions positioned close by, as illustrated in Figure 7f.

It is worth noting that the influence of anions on the hysteresis behavior is not manifest directly. When *V*_g_ is a negative voltage, Cl^–^ ions tend to approach the graphene surface. This increase in the Cl^–^ concentration reduces the interfacial OH concentration, making it challenging to observe the direct effects of Cl^–^. Additionally, SF-GFET exhibits p-type behavior. When *V*_g_ is swept from 0 V to the charge neutrality point at the forward direction, Cl^–^ ions move away from the surface and cations approach it. Under these conditions, the dominance of cation effects further obscures the influence of Cl^–^. This also makes it difficult to detect anion contribution experimentally. Furthermore, the hysteresis of the graphene Dirac point is primarily attributed to the differences in the 2D hydrogen-bond network structure (2D HBNS) of water on the graphene surface, particularly at positive voltages. At these voltages, cations significantly influence the interface, altering the interfacial 2D HBNS. This demonstrates that cations have a much stronger influence on the observed hysteresis effect than do anions.

When the voltage changes from negative to positive, the structured arrangement of the FWL loosens. This results in a notable increase in the number of dangling water molecules.^58^ At low concentrations (1 μM), potassium ions (K^+^) and chloride ions (Cl^–^) form hydration clusters with the dangling water molecules. The abundance of dangling water molecules tends to point toward the surface under electric field.^56^ When the voltage returns from positive to negative, the backward interfacial reorganization evolution would be faster than that of the forward interfacial restructuring process. Correspondingly, the negative interfacial charge in the backward scanning process is more pronounced than that in the forward scanning process. This is consistent with experimental carrier transport results, as a positive hysteresis effect of SF-GFET.

**Figure 8:**
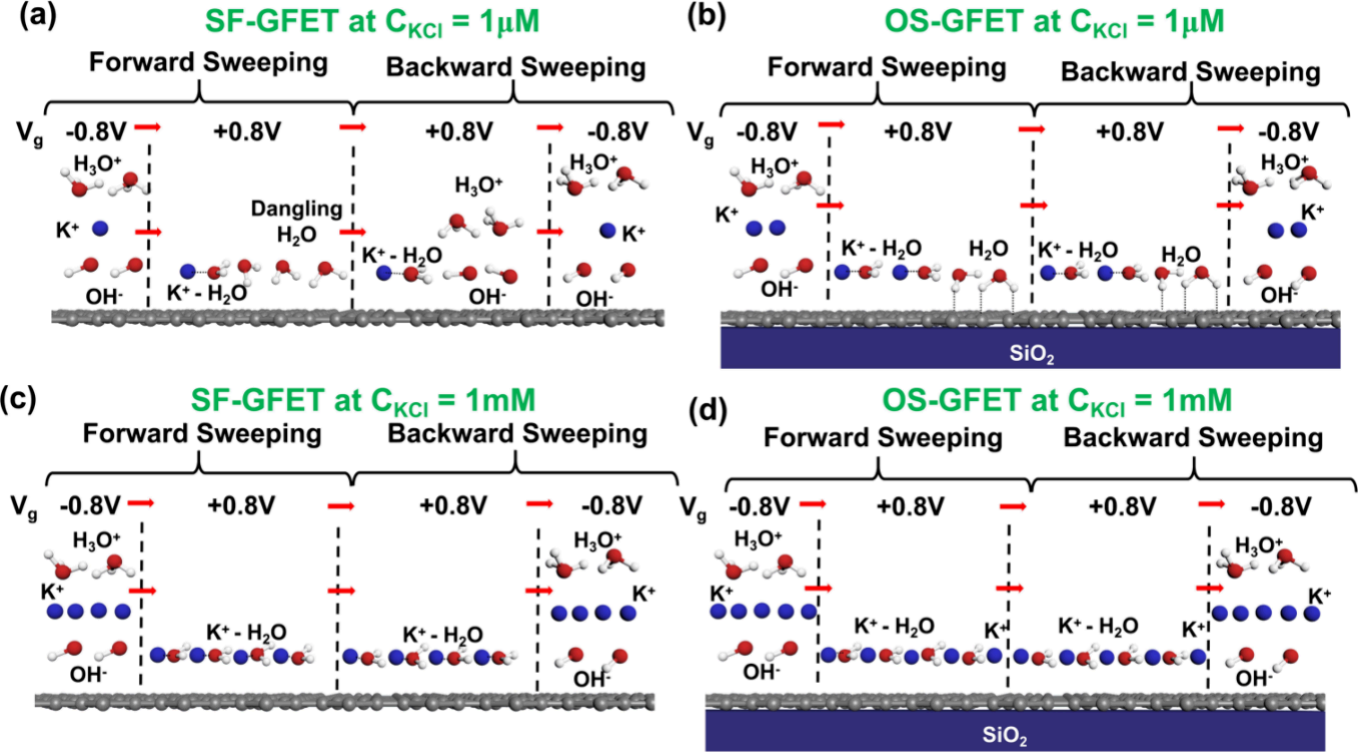
Comparison between dangling molecule evolution of SF-GFET and OS-GFET in a 1 μM and 1 mM KCl environment. For clarity, 2D HBNS and ionic hydration clusters are represented exclusively in an ionic state: (a) SF-GFET with a 1 μM KCl environment. (b) OS-GFET with a 1 μM KCl environment. (c) SF-GFET with a 1 mM KCl environment. (d) OS-GFET with a 1 mM KCl environment.

The comparison between interfacial arrangement under electrical modulation of SF-GFET and OS-GFET with a 1 μM KCl environment is shown in Figure 8a and Figure 8b, respectively. In order to highlight the evolution of dangling water in Figure 8, only part of the ions and molecules are illustrated. As discussed earlier, changes in surface potential significantly impact ion accumulation dynamics. Consequently, within the OS-GFET structure framework, there is an increase in the accumulation of interfacial potassium ions. As discussed earlier, changes in surface potential exert a discernible influence on ion accumulation dynamics. Consequently, within the framework of the OS-GFET structure, the accumulation of interfacial potassium ions increases. During the transition of gate voltage applied to the OS-FET from negative to positive (−0.8 → + 0.8 V), the abundance of dangling water molecules shows preference for pointing toward interface, instead of staying in dangling states. When the gate voltage changes to +0.8 V at backward sweeping, they maintain directionality rather than tending to dissociation and reorientation. This corresponds with the negative hysteresis value of OS-GFET. As the ion concentration increases, the competition between the H-bond structure within water hydration shells and ionic hydration shells become fierce. For SF-GFET in a 1 mM KCl environment, when the gate voltage changes from negative to positive, the dissociation of interfacial water molecules will be suppressed at the graphene–electrolyte interface, and dangling water molecules will form stable configurations under the influence of ion interaction rather than exist in a free state, as shown in Figure 8c. When the ion concentration increases to surface supersaturation, additional ions appear in the SWL.^28^ The specific interfacial evolution process of the SF-GFET interface with 1 mM KCl aqueous solution under electrical modulation is shown in Figure S4. Upon the voltage shifting back from positive to negative, the disparity between the cation–graphene interaction and dehydration energy makes the exchange of cation and anion positions more challenging. Consequently, the positive interfacial charge (K^+^) becomes more pronounced during the backward scanning process compared to the forward scanning process. This aggravates the negative hysteresis effect, as we observed. Similarly, for the OS-GFET, the water molecules oriented toward the substrate are redirected gradually by ions, as shown in Figure 8d. When the gate voltage turns positive, the water molecules’ redirection is inhibited gradually by the ionic layer. Thus, the negative hysteresis value of OS-GFET increases slightly as the concentration rises. The specific interfacial evolution process of the OS-GFET interface with 1 mM KCl aqueous solution is shown in Figure S5.

Discrepancies arise due to the distinctive properties of monovalent and divalent salts (e.g., KCl and MgCl_2_), despite both acting as aqueous structure makers.^28^ Considering the different valences, the quantities of chloride ions vary at equivalent concentrations. Consequently, a magnesium chloride solution contains a greater number of hydrated and structured chloride ions, leading to a difference in the number of captured dangling water molecules at the same concentration. The ionic concentration producing half occupation of MgCl_2_ solution is lower than that of KCl solution. In addition, magnesium ions (Mg^2+^) tend to situate on the outer side of chloride ions (Cl^–^) rather than locate at the interfacial region. Meanwhile, the diffusivity of magnesium ions is relatively lower compared to that of potassium ions at the same region. Thus, at high concentrations, a negative interfacial charge at the interface SF-GFET at the forward scanning process results in a slight reduction of negative hysteresis. For OS-GFET, magnesium ions are positioned closer to the inner side, resulting in a smaller hysteresis value compared to that of SF-GFET with 1 mM MgCl_2_ electrolyte.

### Carrier Storage at the Graphene–Electrolyte Interface

**Figure 9:**
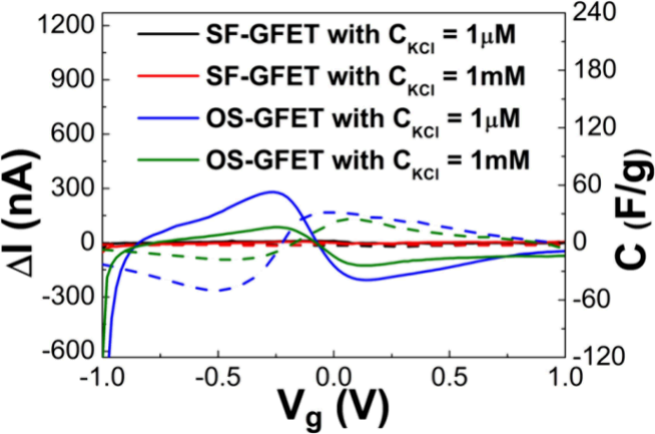
Comparison of the net field-effect current (Δ*I*) and interfacial capacitance (*C*) of SF-GFET and OS-GFET GFET in 1 μM and 1 mM KCl environments. For SF-GFET (hydrophobic surface), the net field-effect current (Δ*I*) is tiny; for OS-GFET (hydrophilic surface), by contrast, it shows peak-current behavior during the gate-voltage scanning process.

We conducted further investigations on the net field-effect current (Δ*I* = *I*_d_ + *I*_s_ + *I*_g_), which indicates carrier storage conditions at the graphene–electrolyte interface. As shown in Figure 9, the Δ*I* of SF-GFET is very small and shows little variation in 1 μM and 1 mM KCl environment. Conversely, in the case of OS-GFET, there is a pronounced peak of Δ*I* behavior with changed gate voltage, and the summit value decreases as the concentration increases. The gate current does not exhibit significant changes within the corresponding voltage range of −0.5 to 0.5 V, as shown in Figure 6d. This indicates carrier accumulation at the solid–liquid interface instead of transportation within graphene in the longitudinal direction. The corresponding capacitances of SF-GFET and OS-GFET in 1 μM and 1 mM KCl environments are also shown in Figure 9. Details of the capacitance calculation follow Δ*Q* = Δ*I* × Δ*t* (as described in Experimental Section). The capacitance of SF-GFET with 1 μM KCl electrolytes is within 4 F/g, and it decreases to 2 F/g as the electrolyte concentration rises to 1 mM (corresponds to 1.49 to 0.75 mF/m^2^, respectively). In contrast, the maximum capacitance value of OS-GFET with KCl electrolytes changes under transient measurement from 53 to 16 F/g (corresponds to 19.8 to 6.08 mF/m^2^) with different ionic concentrations ranging from 1 μM to 1 mM, respectively. This capacitance value of OS-GFET corresponds with previous supercapacitance research.^13^ These show an intuitive observation of the energy storage phenomenon at the interface caused by electrostatic interactions. The performance disparity between SF-GFET and OS-GFET with electrolytes is similar to that with water;^13^ the comparison results are shown in Figure S6. This observation indicates that water molecules play a crucial role in the system. Specifically, the maximum capacitance value of the OS-GFET with water is 56 F/g. This suggests that the addition of ions inhibits the electrostatic charge storage of water molecules at the interface. In the case of SF-GFET, water molecules at the interface are present either in a 2D HBNS structure or as dangling water. The absence of a unified dipole orientation in these water molecules does not help carrier accumulation at the interface, resulting in small interfacial capacitance. Conversely, OS-GFET, with its higher electron affinity, significantly influences the collective dipole behavior of water molecules. This effect also contributes to carrier accumulation at the interface in aqueous solutions. It leads to charge storage within the interface. These findings show the critical role of a hydrophilic interface in the development of supercapacitors. As the ion concentration increases, there is a corresponding increase in the alignment of water molecules directed by ions. The hydration cluster holds the interfacial ions to prevent an aligned dipole structure at the interface. This prevents the formation of a capacitive structure. Simultaneously, the presence of ions elevates the interfacial dielectric constant, making carrier transmission easier and thus leading to a decrease in net current flow.

## Conclusions

In this work, the interfacial arrangement between graphene and a saline solution was experimentally observed for the first time, both with and without surface potential interference. These results are consistent with the conclusions drawn from previous simulation works. When solutions with varying concentrations and different ion valences are exposed, the transformation of ion configurations at the interface can be investigated under different voltage modulations. For SF-GFET with a concentration lower than 1 μM, high interfacial water dissociation, known as 2D HBNS, dominates at the interface. When the concentration increases gradually, the ionic dense adsorption layer competes with the water network structure and gradually plays the dominant role. The competition between the adsorption energy and the hydration tendency causes differences in the arrangement of monovalent and divalent ions at the interface. Differences in the quantity of ions from salt decomposition at equivalent concentrations, e.g., potassium chloride and magnesium chloride, influences the reorientation of surface water molecules. In addition, the arrangement of mono- and divalent ions at the hydrophobic interface exhibits distinct differences. For the OS-GFET, the interfacial water configuration displays collective behavior at low concentrations. However, as the salt solution concentration increases, highly oriented water molecules gradually undergo reorientation. Ionic hydration gradually supplants collective behavior and becomes predominant.

Surface potential and ion concentration are critical factors influencing the formation of interfacial capacitance. For instance, when one side of OS-GFET contacts the ionic solution, carrier storage can be observed significantly, contrasting with minimal carrier storage at the SF-GFET interface. These provide valuable guidance for graphene supercapacitance improvement with a surface potential. On the other hand, interfacial ionic hydration plays a crucial role in the overall capacitance behavior at the interface within the concentration range of micromolar levels. In other words, water molecules rather than ions provide favorable conditions for the formation of a capacitor due to their polarity and high permittivity. Therefore, low ionic electrolytes are preferred to establish a good nanoscopic supercapacitor at the graphene–solution interface. As a consequence, shown in this work, a hydrophobic conducting material with hydrophilic surface interference, e.g., oxide-supported graphene, facilitates the formation of a supercapacitor. Furthermore, this experimental architecture establishes a method for 2D material-liquid fundamental research and guides practical applications.

## Supporting Information Available

(S1) Characteristics of the SF-GFET device and OS-GFET device; (S2) fabrication of the fluidic channel in the SF-GFET device substrate; (S3) electrical results of SF-GFET in a 1 mM potassium chloride aqueous environment and OS-GFET in a 1 μM potassium chloride aqueous environment; (S4) interfacial evolution of SF-GFET in a 1 mM potassium chloride aqueous environment; (S5) interfacial evolution of OS-GFET in a 1 mM potassium chloride aqueous environment; (S6) transportation behavior between SF-GFET and OS-GFET with water and 1 μM KCl (PDF)
