# Supplementary material for: Nanoscopic Supercapacitance Elucidations of the Graphene-Ionic Interface with Suspended/Supported Graphene in Different Ionic Solutions
Source: ACS Appl Mater Interfaces. 2025 Jan 13;17(3):5419–29. doi: 10.1021/acsami.4c16362 (PMC11758774; doi:10.1021/acsami.4c16362)
Supplement: Supplementary file 1 — am4c16362_si_001.pdf [file am4c16362_si_001.pdf]

Supporting Information

Nanoscopic Supercapacitance Elucidations of Graphene-Ionics Interface with Suspended/Supported Graphene in Different Ionic Solutions

Yu-Xuan Lu[1], Ming-Hsiu Tsai[1], Cheng-Yu Lin[1], Wei-Yen Woon[2], Chih-Ting Lin[1,3]*

[1]Graduate Institute of Electronics Engineering, National Taiwan University, Taipei 10617, Taiwan

[2]Department of Physics, National Central University, Jungli 32054, Taiwan.

[3]Graduate School of Advanced Technology, National Taiwan University, Taipei 10617, Taiwan

*Email: timlin@ntu.edu.tw

KEYWORDS: Graphene Supercapacitor, Suspended Graphene, Graphene-Ionic Interface, Solid-Liquid Interface, Interfacial Water Arrangement

S1. The characteristics of SF-GFET device and OS-GFET device

To further demonstrate the suspended graphene, we additionally take SEM image when SF-GFET is placed upright and tilted at a 60-degree angle from the horizontal, as illustrated in Fig. S1(a). The suspended flat graphene sheet shows that the SF-GFET structure satisfies the model requirements. The corresponding optical image to the Raman spectroscopy of SF-GFET and OS-GFET are demonstrated in Fig. S1(b). The optical image displays a clear discrimination to identify the integral presence of graphene. The prominent characteristics in Raman spectrum are the G peak around 1580 cm^{-1} and the 2D band around 2700 cm^{-1}; the typical features prove the existence of graphene.

We further conducted regional measurements to validate the quality of large-area graphene. For SF-GFET, characteristics measurements were performed over a large area, encompassing 20 points evenly spaced at intervals of 40 μm. The average results indicate an I_D/I_G ratio of 0.05, an I_{2D}/I_G ratio of 1.14, and a FWHM of the I_G peak of 26.33 cm^{-1}. For OS-GFET, Raman measurements were conducted at 8 selected points across the device, covering the top-left, top-right, bottom-left, bottom-right, and central regions. The averaged results reveal an I_D/I_G ratio of 0.04, an I_{2D}/I_G ratio of 1.52, and a FWHM of the I_G peak of 20.32 cm^{-1}. These findings provide additional insights into the structural homogeneity and crystallinity of the graphene. This detailed information has been incorporated into both the main text and the supplementary material to strengthen the discussion and clarify the methodology.

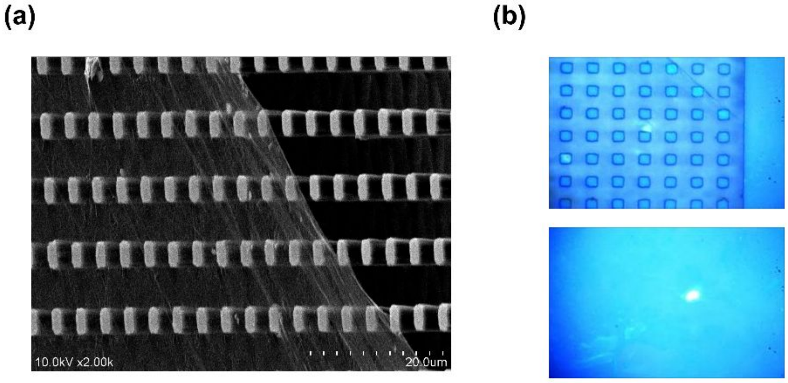

Figure S1: (a) SEM image of SF-GFET tilted at a 60-degree angle from the horizontal; (b). The corresponding optical image to the Raman spectroscopy of SF-GFET (above) and OS-GFET (below).

S2. The fabrication of fluidic channel in SF-GFET device substrate

The detailed fabrication process of SF-GFET is illustrated in Fig. S2, which mainly consists of bilayer graphene and suspended substrate fabrication. Bilayer graphene fabrication is a critical process. The monolayer graphene film is grown on the copper foil by chemical vapor deposition (CVD) and then separated by electrical bubble delamination with the support of PMMA protective film. To establish the bilayer graphene, physically stacking the monolayer graphene together in deionized (DI) water environment and removing the water by spinning and baking. Further overlay and separation also rely on the support of the PMMA sheet. To fabricate the microchannel structure, on the other hand, spinning photoresist onto a SiO_2 (300 nm) / Si substrate and generating microfluidic pattern by photolithography. After dry etching the SiO_2

with thickness of 300 nm and Si layers with depth of 30 μm, the microfluidic trench can be used to store aqueous solution. Then, Au electrodes adjacent to the microfluidic channel are deposited through evaporation. Afterward, bilayer graphene film is transferred across the trench. Finally, immersing SF-GFET in acetone for 24 hours to remove the PMMA layer. Then, the device is submersed in isopropanol (IPA) and DI water for 5 minutes to cleanse any residues.

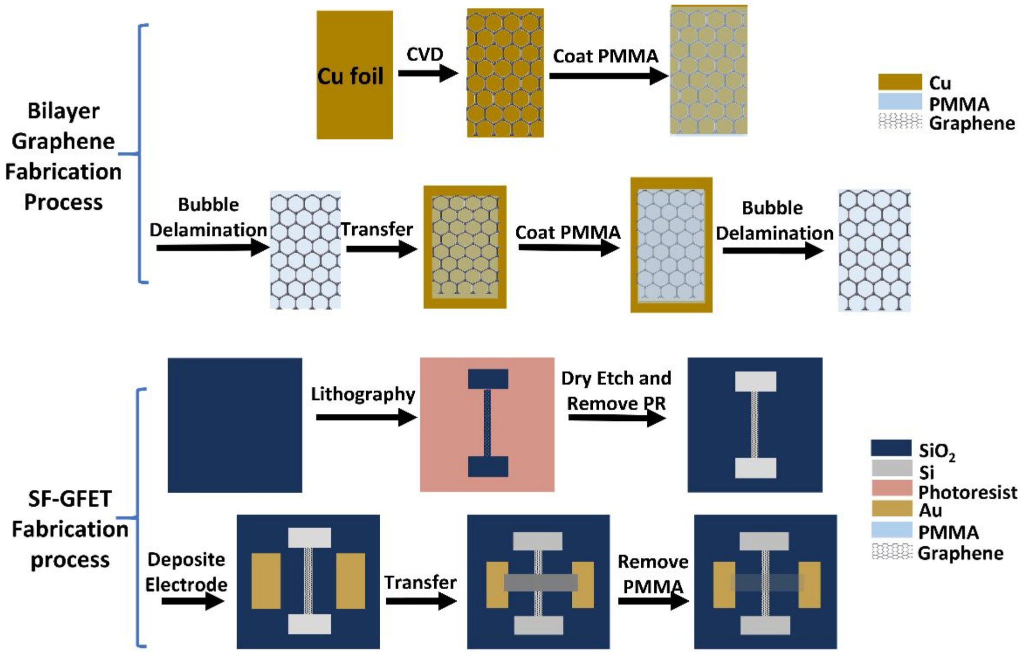

Figure S2. The fabrication process of bilayer graphene and SF-GFET device.

S3. Electrical Results of SF-GFET at 1 mM Potassium Chloride Aqueous Environment and OS-GFET at 1 μM Potassium Chloride Aqueous Environment

The hysteresis behavior through the graphene field-effect current (I_d) of SF-GFET in one-side electrolyte solution as 1 mM KCl aqueous solution with different gate voltage step rates are shown in Fig. S3(a), and the electrical results of OS-GFET with 1 μM KCl aqueous solution is shown Fig. S3(b). The corresponding conducting path through gate current (I_g) with different gate voltage step rates shown in Fig. S3(c) and Fig. S3(d) respectively. In these figures, I_d varies throughout the V_g scanning process, reflecting dynamic changes in the interfacial configuration. This variation in I_d is not significantly correlated with changes in I_g, indicating that the interfacial capacitive

gating effect primarily drives channel doping. These changes reflect the interfacial arrangement.

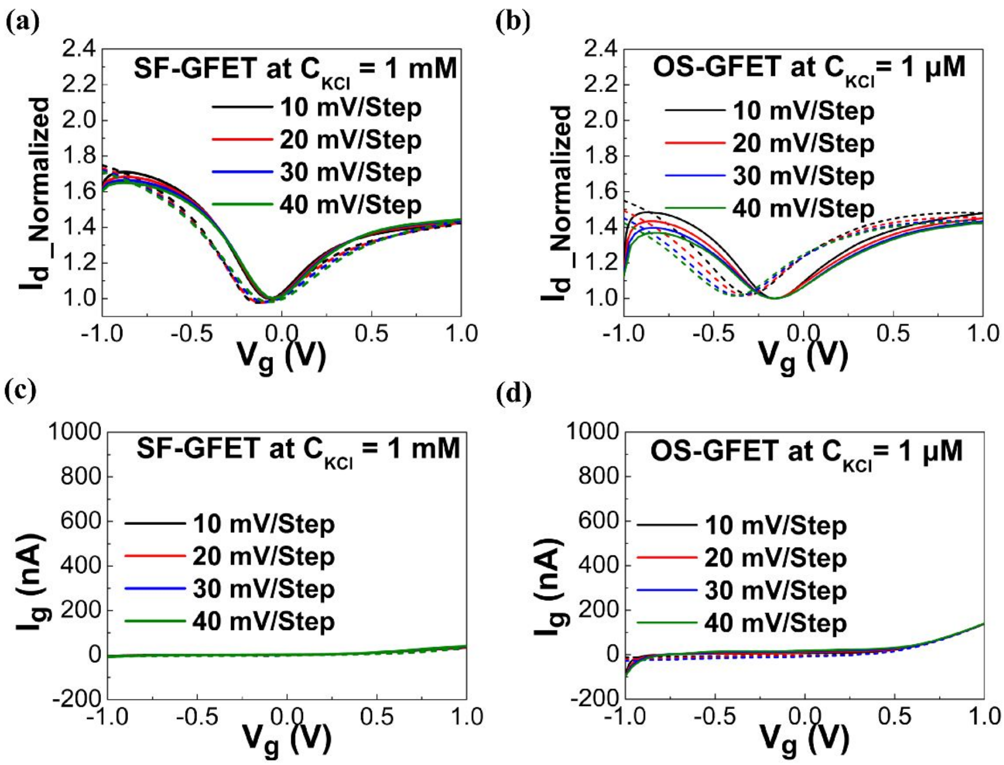

Figure S3. Drain current behavior (I_d) with changed gate voltages and different step rates: (a) SF-GFET contact with 1 mM KCl electrolyte solution; (b) OS-GFET contact with 1 μM KCl electrolyte solution. Gate current behavior (I_g) with changed gate current and different step rate: (c) SF-GFET contact with 1 mM KCl electrolyte solution; (d) OS-GFET contact with 1 μM KCl electrolyte solution.

S4. Interfacial Evolution of SF-GFET at 1 mM Potassium Chloride Aqueous Environment

In specific terms, the gate voltage is initially set at -1 V, generating a substantial electric field directed away from the surface. At this stage, Cl^- hydration shells approach the graphene surface, while K^+ hydration shells move away from it, both residing within the first water layer. With an increase in salt concentration, the concentration of ions at the graphene-electrolyte interface rises, resulting in a denser hydration shell at the interface [24]. The consumption of dangling water molecules weakens the 2D HBNS, leading to a relatively lower dissociation of water molecules compared to low concentrations. Consequently, this leads to a more negatively charged interface, inducing hole doping of graphene, as illustrated in Fig. S4(a).

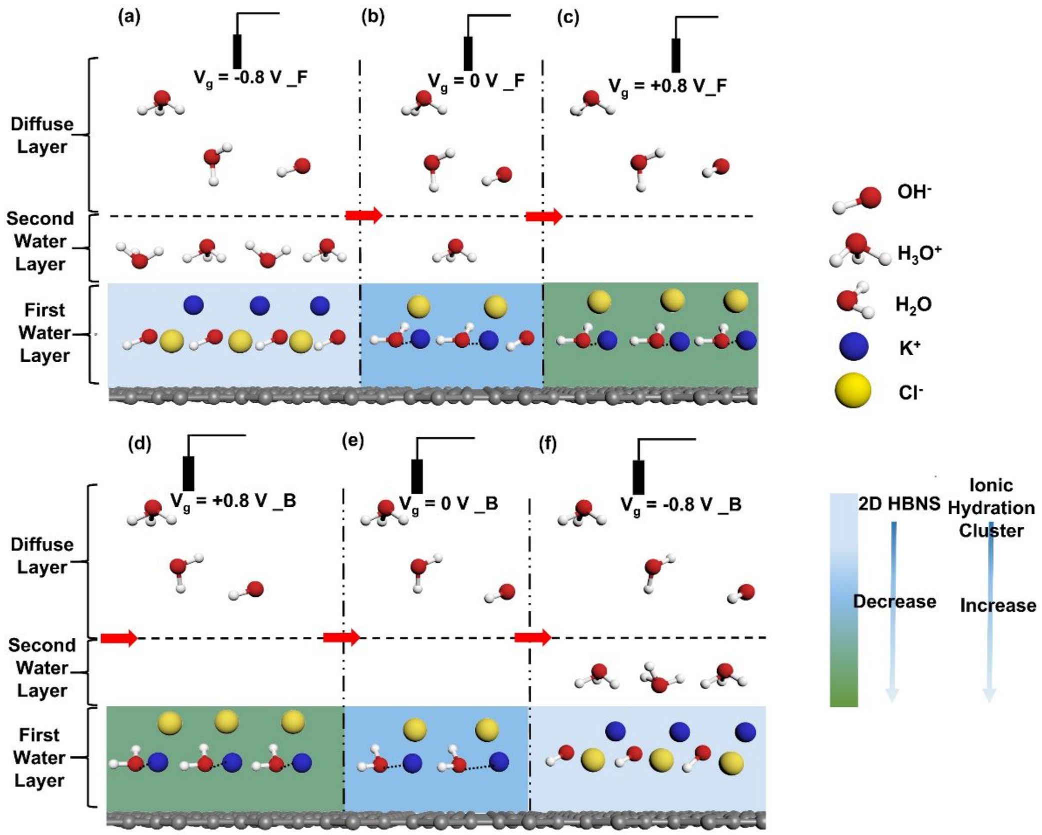

Figure S4. Schematic of interfacial evolution of SF-GFET in 1 mM KCl electrolyte solution with different applied gate voltages. The right color bar shows the population degree of the interfacial water configuration in the first water layer. The 2D HBNS (light blue) dominates the interfacial water configurations for $V_g = -0.8$ V. On the other hand, the ionic hydration cluster structure (green) dominates the interfacial configuration for $V_g = +0.8$ V. Similarly, 2D HBNS and ionic hydration clusters are exclusively represented in their ionic states for clarity. (a) $V_g = -0.8$ V at the forward scan path. (b) $V_g = 0$ V at the forward scan path. (c) $V_g = +0.8$ V at the forward scan path. (d) $V_g = +0.8$ V at the backward scan path. (e) $V_g = 0$ V at the backward scan path. (f) $V_g = -0.8$ V at the backward scan path.

As the V_g scans towards 0 V, the negative electric field decreases, as shown in Fig. S4(b). Cl^- move outwards from the interface, forming hydration groups with more external water molecules, while K^+ hydration shells dehydrate and move towards the interface. Simultaneously, the weakening of the 2D HBNS continues with decreasing voltage, resulting in a less negative interface charge. As shown in Fig. S4(c), upon further scanning of V_g to a more positive voltage, K^+ hydration shells rearrange at the interface, and Cl- locate at the inner side of the first water layer. At the same time, 2D

HBNS weakens in further step, causing water molecules dehydrated from 2D HBNS and subsequently hydrate with surrounding ions. Additionally, due to the Cl^- residing on the outskirt of the first water layer and neighboring the second water layer, it is hydrated by a great number of water molecules from all directions. The hydration structure at the interface is more stable. These result in graphene electronic doping. At the initiation of the backward scanning process, the interfacial configuration closely resembles that at V_g = +0.8 V at the forward sweeping process, as shown in Fig. S4(d). As the V_g moves back to 0 V, as shown in Fig. S4(e), K^+ and Cl^- hydration shells dehydrate slightly and 2D HBNS strengthens. Insufficiency of dangling water molecules hinders 2D HBNS formation. Nonetheless, this structure still induces electronic doping. Finally, V_g turns to a more negative value, as shown in Fig. S4(f). Cl^- hydration shells dehydrate and move approach the graphene. The strong hydration shells around Cl^- impede the dissociation process, hindering the formation of the 2D HBNS. Consequently, the dissociation rate is lower than that observed during the forward scan, and the reverse-path hole current is below the forward-path hole current.

S5. Interfacial Evolution of OS-GFET at 1 mM Potassium Chloride Aqueous Environment

Fig. S5 illustrates the schematic of the interfacial evolution of OS-GFET in 1 mM potassium chloride aqueous environment during the gate-voltage scanning process. At the start of the forward scanning path, the negative electric field results in the formation of 2D HBNS structure at the interface [50]. The hydration structure of Cl^- and K^+ are arranged within the first water layer, as shown in Fig. S5(a). Thus, graphene is hole-doped at this step. When V_g transitions towards 0 V, 2D HBNS gradually disintegrates. Plenty of water form hydration cluster around ions at the inner layer, as shown in Fig. S5(b). Excessive dangling water points toward the hydrophilic surface gradually. The number of dangling water diminishes concomitantly with an increase in ion concentration. When V_g further turns to a more positive voltage, water molecules enhances the hydrogen-bonding networks. I_d turns to the electron type, and the surface becomes electron-doped, as shown in Fig. S5(c). In the range of +0.6 V to +1 V, I_g experiences a significant increase. The gate voltage changes with the scanning step, demonstrating that the electric double-layer capacitance dominates the leakage current. On the hydrophilic surface, as depicted in Fig. S5(d), the interfacial water molecules exhibit a preference for adopting a configuration pointing towards ions and surface, graphene keeps electron type when gate voltage changes from positive to 0 V. When the gate voltage (V_g) continues to shift towards -1 V, the interfacial water molecules undergo a reconfiguration into the 2D hydrogen-bonded network structure. This reconfiguration results in the reintroduction of hole doping in the graphene, as depicted

in Fig. S5(f). Consequently, this results in a negative hysteresis effect in the case of OS-GFET.

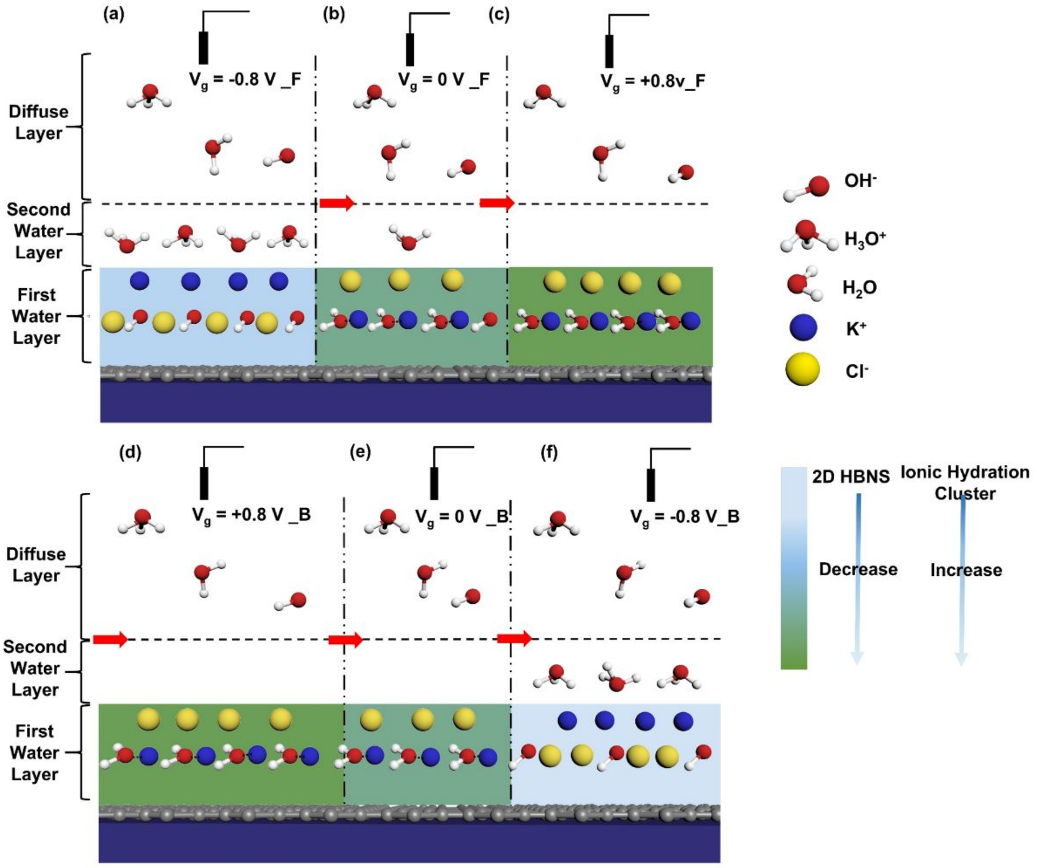

Figure S5. Schematic of interfacial evolution of OS-GFET in 1 mM KCl electrolyte solution with different applied gate voltages. The right color bar shows the population degree of the interfacial water configuration in the first water layer. The 2D HBNS (light blue) dominates the interfacial water configurations for $V_g = -0.8$ V. On the other hand, the ionic hydration cluster structure (green) dominates the interfacial configuration for $V_g = +0.8$ V instead of pointing -toward the surface water dipole at 1 μM KCl. Similarly, 2D HBNS and ionic hydration clusters are exclusively represented in their ionic states for clarity. (a) $V_g = -0.8$ V at the forward scan path. (b) $V_g = 0$ V at the forward scan path. (c) $V_g = +0.8$ V at the forward scan path. (d) $V_g = +0.8$ V at the backward scan path. (e) $V_g = 0$ V at the backward scan path. (f) $V_g = -0.8$ V at the backward scan path.

S6. Transportation behavior between SF-GFET and OS-GFET with water and 1 μM KCl

To explore the interfacial transformation of graphene with addictive ions to water at different states, we measure the graphene field-effect current (I_d) of SF-GFET and OS-GFET with water and 1 μM KCl electrolyte solution, as shown in Fig. S6(a). For SF-GFET, the existence of Cl^- at the interface results in the hole-doping of graphene. While due to the concentration of accumulated ions at the interface, no significant change in hysteresis value is observed. For OS-GFET, hysteresis value decreases from -0.18 V to -0.15 V. Hydrophilic surface adsorbs more collected ions at the interface, this influences the water dynamic dissociation process. In addition, reoriented dangling water by hydration cluster impedes hydrolysis at the interface.

The net field-effect current (ΔI) reflects the interfacial water evolution at the graphene-electrolyte interface, this correspond with interfacial capacitance (C). As depicted in Fig. S6(b), ΔI of SF-GFET. This indicates that the hydrophobic characteristic of the intrinsic graphene surface holds the interfacial configuration, and carrier transfer is present at the interface. In contrast, for OS-GFET, ΔI exhibits quite an apparent fluctuation. With the addition of ions, the peak current value drops from 297 nA to 280 nA. The corresponding capacitance drops from 56 F/g to 53 F/g. Due to the charge accumulation within the local area, which may relate to the interfacial collective dipole behavior, the decrease in peak current value may demonstrate water reorientation.

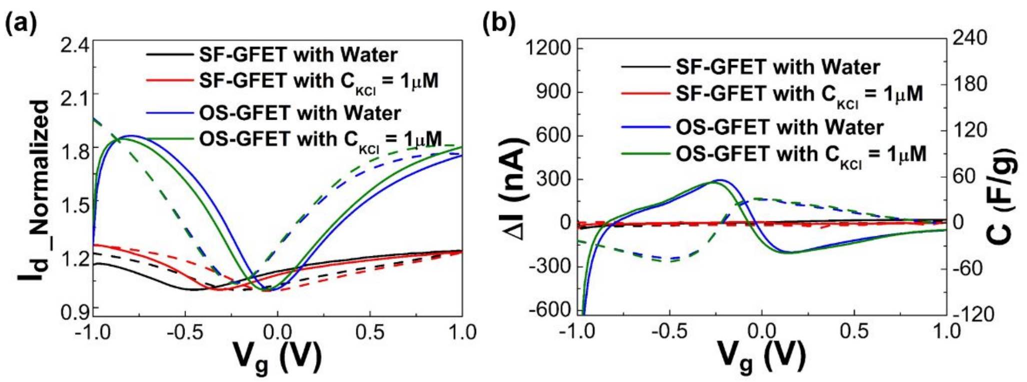

Figure S6. Transportation behavior comparison between SF-GFET and OS-GFET with water and 1 μM KCl solution: (a) Drain current behavior (I_d) of SF-GFET and OS-GFET with water and 1uM KCl electrolyte solution; (b) The net field-effect current

(ΔI) and interfacial capacitance (C) of SF-GFET and OS-GFET GFET in water and 1uM KCl electrolyte solution.
